# Retraction: Evaluation of 'Normal' Cognitive Functions and Correlation With MRI Volumetry: Towards a Definition of Vascular Cognitive Impairment

**DOI:** 10.7759/cureus.r219

**Published:** 2026-01-28

**Authors:** Uma Sundar, Amita Mukhopadhyay, Sheelakumari Raghavan, Ipsita Debata, Ramshekhar N Menon, Chandrasekharan Kesavadas, Nilesh Shah, Balkrishna B Adsul, Anagha R Joshi, Janardhan Tejas

**Affiliations:** 1 Department of Medicine, Lokmanya Tilak Municipal Medical College and General Hospital, Mumbai, IND; 2 Department of Hospital and Health Management, Institute of Health Management Research Bangalore, Bengaluru, IND; 3 Department of Radiology, Sree Chitra Tirunal Institute for Medical Sciences and Technology, Thiruvananthapuram, IND; 4 Department of Community and Family Medicine, Kalinga Institute of Medical Sciences, Bhubaneswar, IND; 5 Department of Neurology, Sree Chitra Tirunal Institute for Medical Sciences and Technology, Thiruvananthapuram, IND; 6 Department of Psychiatry, Lokmanya Tilak Municipal Medical College and General Hospital, Mumbai, IND; 7 Department of Community Medicine, Hinduhrudaysamrat Balasaheb Thackarey Medical College and Dr RN Cooper Municipal General Hospital, Mumbai, IND; 8 Department of Radiology, Lokmanya Tilak Municipal Medical College and General Hospital, Mumbai, IND; 9 Department of Forensic Medicine and Toxicology, Karpaga Vinayaga Institute of Medical Sciences and Research Center, Chengalpattu, IND

This article has been retracted by the Editor-in-Chief due to a copyright violation by the authors concerning the unauthorized use of the Mini-Mental State Examination (MMSE). As a result, this article has been retracted and the article contents have been removed.

